# Reconstruction of displaced acromio-clavicular joint dislocations using a triple suture-cerclage: description of a safe and efficient surgical technique

**DOI:** 10.1186/1754-9493-6-25

**Published:** 2012-10-25

**Authors:** Gunther H Sandmann, Frank Martetschläger, Lisa Mey, Tobias M Kraus, Arne Buchholz, Philipp Ahrens, Ulrich Stöckle, Thomas Freude, Sebastian Siebenlist

**Affiliations:** 1 Department of Traumatology, Klinikum rechts der Isar, Technical University Munich, Ismaninger Str. 22, Munich D- 81675, Germany; 2 Berufsgenossenschaftliche Unfallklinik Tuebingen, Schnarrenbergstr. 9, Tuebingen D- 72076, Germany

**Keywords:** Acromioclavicular joint, Dislocation, Rockwood, Cerclage

## Abstract

**Purpose:**

In this retrospective study we investigated the clinical and radiological outcome after operative treatment of acute Rockwood III-V injuries of the AC-joint using two acromioclavicular (AC) cerclages and one coracoclavicular (CC) cerclage with resorbable sutures.

**Methods:**

Between 2007 and 2009 a total of 39 patients fit the inclusion criteria after operative treatment of acute AC joint dislocation. All patients underwent open reduction and anatomic reconstruction of the AC and CC-ligaments using PDS® sutures (Polydioxane, Ethicon, Norderstedt, Germany). Thirty-three patients could be investigated at a mean follow up of 32±9 months (range 24–56 months).

**Results:**

The mean Constant score was 94.3±7.1 (range 73–100) with an age and gender correlated score of 104.2%±6.9 (88-123%). The DASH score (mean 3.46±6.6 points), the ASES score (94.6±9.7points) and the Visual Analogue Scale (mean 0.5±0,6) revealed a good to excellent clinical outcome. The difference in the coracoclavicular distance compared to the contralateral side was <5 mm for 28 patients, between 5-10 mm for 4 patients, and more than 10 mm for another patient. In the axial view, the anterior border of the clavicle was within 1 cm (ventral-dorsal direction) of the anterior rim of the acromion in 28 patients (85%). Re-dislocations occured in three patients (9%).

**Conclusion:**

Open AC joint reconstruction using AC and CC PDS cerclages provides good to excellent clinical results in the majority of cases. However, radiographically, the CC distance increased significantly at final follow up, but neither the amount of re-dislocation nor calcifications of the CC ligaments or osteoarthritis of the AC joint had significant influence on the outcome.

**Level of evidence:**

Case series, Level IV

## Introduction

Regarding acromioclavicular (AC) joint dislocations, conservative therapy is widely accepted as gold standard for type I and II injuries according to Rockwood et al. [1]. There is also consent that type IV to VI dislocations should usually be treated surgically [2-4]. Conservative treatment of grade III AC joint dislocations is widespread and several promising results have been published [5,6]. However, some authors have reported residual symptoms like pain and weakness after conservative treatment of grade III injuries. Therefore, they recommend operative reconstruction in young, athletic and ambitious patients [7-9].

Several clinical studies reported promising results for different open surgical techniques ranging from the reconstruction of the deltoid/ trapezoid fascia [10] to the use of a hook plate [11], temporary K- wires [12], CC- screw [13] or primary ligament transfers [14]. In recent years, arthroscopic procedures using suture anchors or tight rope fixation with or without tendon grafts are being advocated more and more frequently [4,15-17].

The cerclage augmentation of the coracoclavicular (CC) ligaments has been shown to produce good to excellent clinical results, while the radiological results showed a certain amount of re-dislocation, independently from the type of sutures used [3,18,19]. The presentedtechnique, using AC and CC cerclages with resorbable sutures (PDS®; Ethicon, Norderstedt/Germany) can provide an anatomic restoration of the joint congruity and might lead to improved AC stability. However, data regarding this operative technique is limited and little is known about additional factors influencing the surgical outcome [19].

Therefore, the purpose of the present study was to evaluate the functional and radiological outcome after triple cerclage fixation for anatomic reconstruction of acute AC-joint dislocations Rockwood type III to V with resorbable sutures [1,19].

We hypothesized that this technique will lead to equal or superior functional results when compared to those reported in the literature for other surgical procedures and that the additional AC cerclage improves the radiological results with a lower rate of re-dislocation. Furthermore, we postulated that the type of injury would not influence the functional outcome.

## Materials and methods

### Patients

For this retrospective data analysis we evaluated the records of all patients operatively treated due to AC joint dislocation Rockwood [1] type III to V between 2007 and 2009 in a level 1 trauma centre. In all cases an anatomic reconstruction of the CC and the AC ligaments using cerclages with resorbable sutures was performed. For inclusion in the study, which was approved by an ethic committee, all patients had to declare their written consent to participate. Furthermore the patients had to be skeletally mature and at least 2 years out of surgery. Patients with chronic AC-joint dislocation (delay from injury to surgery of more than 21 days), previous AC- joint surgery, and/or associated fractures of the clavicle, acromion or the coracoid process were excluded from the study. Of the 39 patients that met the inclusion criteria, 33 (85%) were available for clinical evaluation. From these 33 patients three refused the x-ray at follow up. The average patient age was 39 years (range, 18–71 years) at time of surgery. According to the Rockwood classification [1] there were nine type-III, nine type-IV and fifteen type-V injuries in 30 male and 3 female patients (see Table 1). In 19 patients the dominant arm was affected. Twenty-five patients had suffered an isolated injury of the AC-joint. Three patients had additional ipsilateral rib fractures, one patient had a fracture of the contralateral clavicle, and another patient had a concomitant type B fracture of the pelvis.

**Table 1:** Patient characterization and clinical outcome

	
Number of patients at follow up	33/39 (85%)
Mean age (range)	39 (18-71)
Gender N (%)	Women: 3 (9%)
	Men: 30 (91%)
Mean time between injury and surgery, days (range)	5 (0-9)
Dominant shoulder N (%)	20 (61%)
BMI [kg/m2] (range)	25.3 (21-34)
Mean duration of surgery, minutes (range)	76 (55-111)
Subjective satisfaction N (%)	
Excellent	20 (61%)
Good	11 (33%)
Fair	1 (3.0%)
Poor	1 (3.0%)
Mean CS at FU ± SD (range)	94.3 ± 7.1 (73-100)
Results N (%):	
Excellent	26 (79%)
Good	6 (18%)
Satisfactory	1 ( 3%)
Fair	0
Mean DASH score points ± SD (range)	3.46 ± 6.6 (0-32)
Number of working patients N (%)	30 (91%)
Mean of additional DASH working module ± SD (range)	1.5 ± 3.8 (0-19)
Number of athletic patients N (%)	20 (61%)
Mean of additional DASH sports module	7.2 ± 11.1 (0-37.5)
ASES score ± SD (range)	94.6 ± 9.7 (64-100)
VAS ± SD (range)	0,5 ± 0,6

The predominant injury mechanisms were bicycle/motorbike accidents in thirteen patients and falls during skiing or snowboarding in eight patients. Thirty patients were employed and twenty patients were previously engaged in recreational sporting activities.

### Surgical management

**Figure 1:**
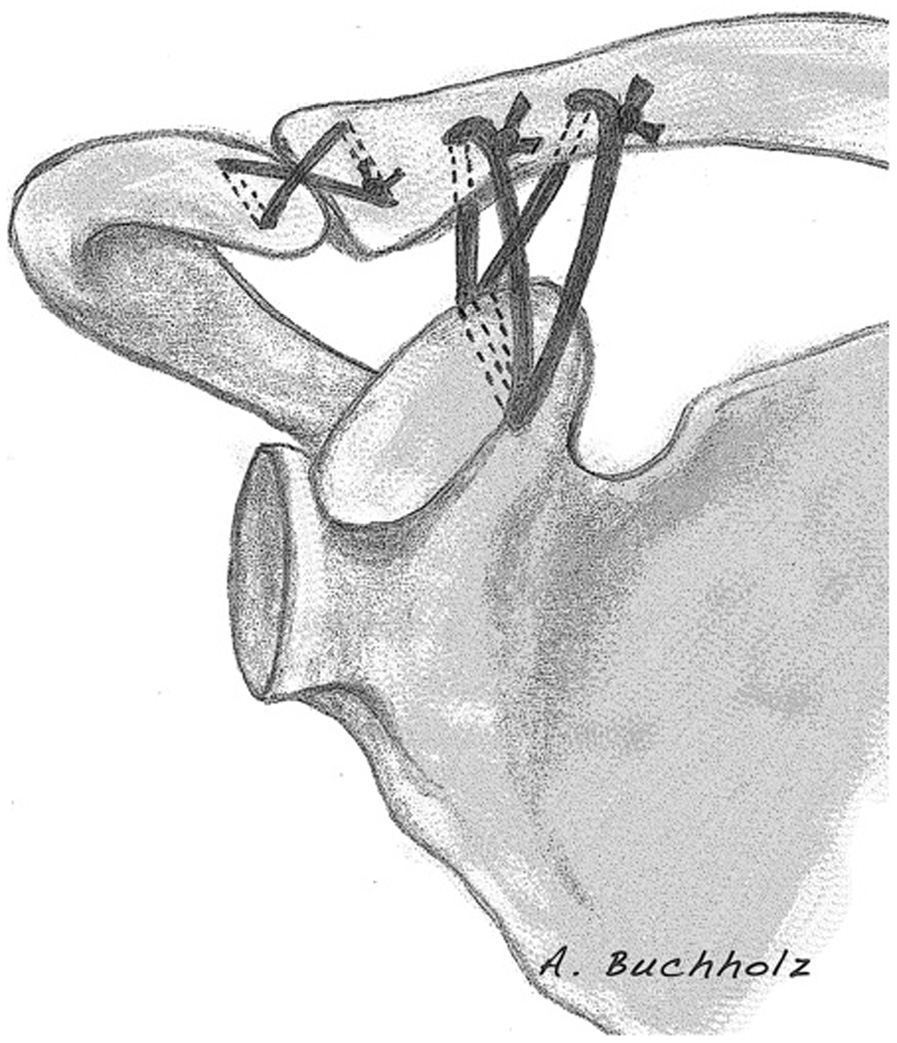
Scheme of the triple cerclage technique.

The mean interval between injury and operation was 5 days (range 0–9 days). General anaesthesia was used in all cases and patients were placed in “beach-chair” position. An anterior approach using a vertical skin incision medial to the AC-joint (“saber cut”) was performed in all cases. After exposure of the coracoid process, a subcoracoid passage of two 1.5 mm PDS-cords was performed using a Deschamps. Next, two drill holes are made into the clavicle, considering the anatomic insertions of the CC ligaments [20]. The reduction was performed under visual and fluoroscopic control and the sutures were tightened after transosseous passage of the clavicle. An anatomic reduction of the joint was performed, though published data have shown a an elongation of the PDS material [2,21-23]. In addition, the AC ligament complex was reconstructed by use of a transosseous 1.0 mm PDS® cerclage (see Figure 1). Finally, the trapezoid/deltoid fascia was reconstructed and torn disci in the AC- joint were removed.

For postoperative management, the shoulder was immobilized in a sling (Medisling®, Medi, Bayreuth, Germany) for six weeks. Passive and active (gravity-assisted) exercises out of sling were started under physiotherapists supervision two days after surgery limiting the range of motion to 90° of shoulder flexion and abduction. Six weeks after surgery, the sling was removed and full range of motion allowed. Heavy weight lifting and overhead sporting activities were restricted for 12 weeks postoperatively.

### Evaluation

At time of follow-up all patients were invited for clinical evaluation. Personal interviews and physical examinations were carried out by an independent investigator (L.M), not involved in the patients initial treatment. The Constant- Murley score [24], the DASH Score, the ASES score, the Simple Shoulder Test and the VAS were recorded.

**Figure 2:**
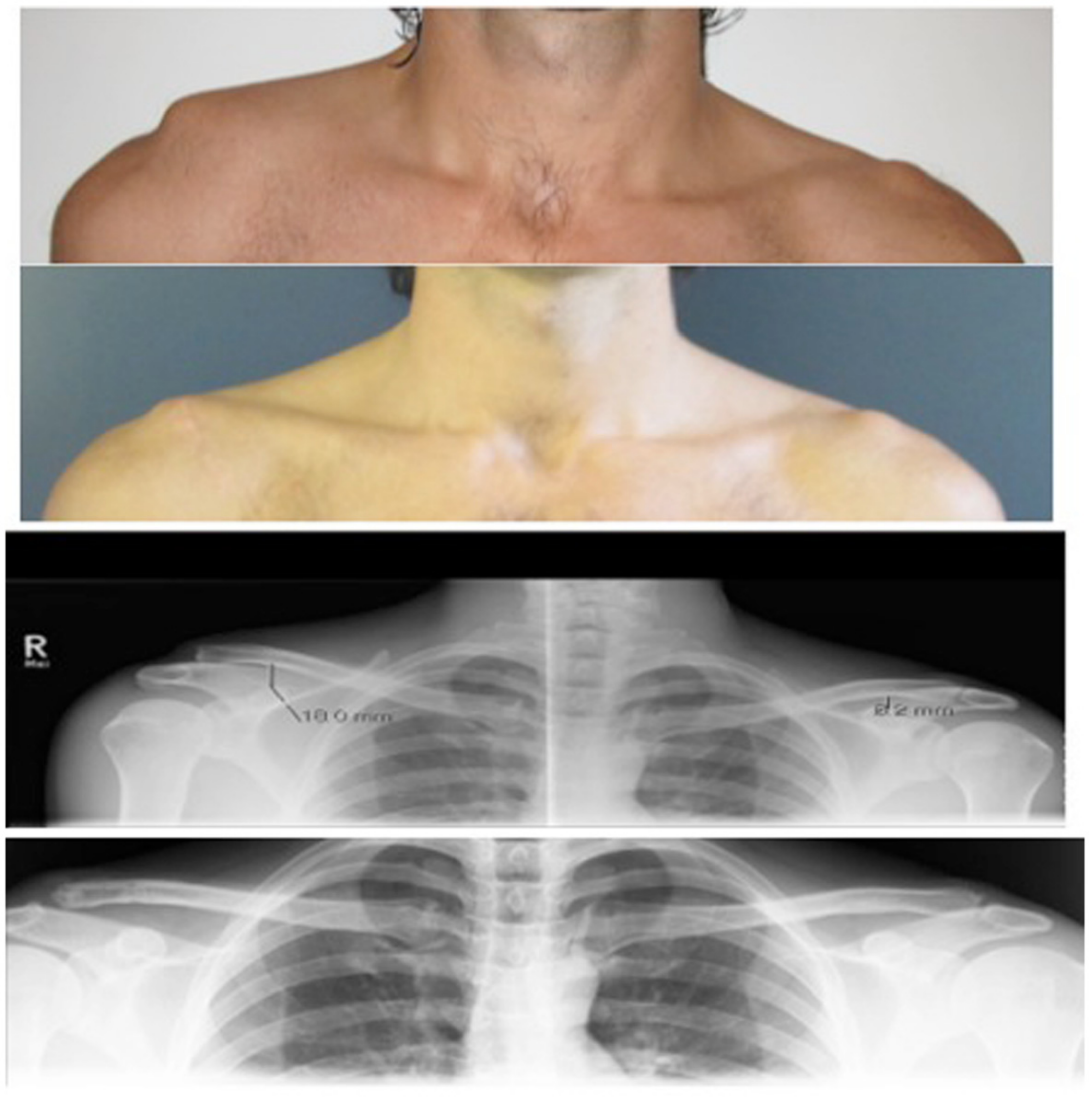
Clinical and radiological image of the AC- joint dislocation, Rockwood III before and 9 months after surgery.

The radiological evaluation included weighted shoulder radiographs (10 kg each side) in anterior-posterior (A-P) direction (“panorama view”, see Figure 2) pre-operatively, post-operatively and at time of follow-up. Additionally, we performed axial radiographs of the operated shoulder to describe the vertical and the A-P translation of the AC-joint. According to Hessmann et al. [25] the radiographs were analyzed by measuring the CC distance (CCD = distance between the inferior rim of the clavicle and the superior rim of the coracoid) on both shoulders. Moreover, the vertical AC distance (vACD = distance between the superior rim of the clavicle and the acromion) was evaluated. On the axial view, the horizontal acromio-clavicular distance, meaning the distance between the anterior rim of the clavicle and the anterior border of the acromion, was measured. The congruity of the AC joint, the presence of degenerative changes of the AC joint, and periarticular ossifications were investigated by three authors independently (GHS, FM, SS). CC ligament calcifications were classified as absent, minor (i.e. spots or small ossicles) and major (i.e. almost complete bridging between the coracoid process and the clavicle) [18].

### Statistical analysis

Statistical analysis was performed by an independent data analyst using the SPSS version 13.0 software (SPSS, Chicago, Illinois). The Mann–Whitney-U test was used for the qualitative data analysis. The significance level was set as *p* ≤ 0.05.

## Results

33 of the 39 included patients (85%) were available for follow up and returned for clinical and radiological assessment. From the 33 patients six refused the x-ray at follow up. The mean duration of follow up was 32 months (range 24–58 months).

### Clinical outcome

On self-evaluation, 31 patients (94%) graded their functional results as excellent or good, and two graded their results as fair and poor. The results of the self-evaluation could be confirmed by the results in the different evaluated shoulder scores (see Table 1).

The mean absolute Constant-Murley score (CS) [24] was 94.3 ± 7.1 (range 73–100) with an age and gender correlated Constant score (agCS) of 104.2% ± 6.9 (88- 123%). According to Boehm et al. [26] these results were subdivided in the following categories: twenty-six excellent results (100–91 points), six good results (89–80 points) and one satisfactory result (79–70 points). There was no significant correlation between the agCS and the grade of injury according to the Rockwood classification (*p* = 0.39). The agCS was 103.2% for Rockwood type III injuries, 101.7% for Rockwood type IV injuries and 103,9.3% for Rockwood type V injuries.

The mean DASH score was 3.46 ± 6.6 points, and 18 patients reached the maximum score of 0 points. The additional working module (30 of 33 were at work) was 1.5 ± 3.8 points and twenty-three patients (82%) reached the maximum of 0 points.

Twenty patients regularly participated in sports and reached mean 7.2 ± 11.1 points in the DASH sports module. Of these patients, 10 (50%) reached their pre-injury sports activity level at a mean of 17 weeks with the maximum amount of 0 points.

The mean ASES score was 94.6 ± 9.7, and 17 patients (52%) reached the maximum of 100 points. Evaluating the Simple Shoulder Test (SST), we found that six patients were not able to carry a weight of 9 kg on the injured site. Additionally, four patients could not sleep on the affected side and four patients were not able to throw overhead.

On visual analogue scale twenty-five patients had no pain at all, five patients had very slight pain (i.e. VAS=1) and two patients had intermittently a medium pain revealing a value of 5, a regular pain medication was denied. The mean VAS value was 0.5 ± 0.6.

Clinical examination showed no limitation of the range of motion in abduction, flexion, external and internal rotation compared to the contralateral shoulder. The BMI was on average 25.3 kg/m^2^ and twenty patients had normal body weight while thirteen patients were overweight with a BMI >25 kg/m^2^. There was no significant correlation between the BMI and the functional outcome measured by the age and gender correlated Constant score (p=0.79).

### Radiological outcome

For radiological evaluation, the pre-operative, the post-operative and the x-rays obtained at time of follow-up were reviewed (see Table 2).

**Table 2:** Radiological results

	
Mean CC distance before surgery (mm), injured side	19.1 ± 4.18
Mean CC distance after surgery (mm), day 2 after surgery	9.0 ± 3.84
Mean CC distance at last follow-up (mm), injured side	12.2 ± 3.12
Mean CC distance (mm), unaffected side	9.7 ± 1,84
P-value between injured side at follow-up and unaffected side	< 0.001
N of patients (%) with CC distance in comparison to the contra-lateral side	
< 5 mm	28 (85%)
5-10 mm	4 (12%)
> 10 mm	1 (3%)
Vertical AC distance (mm) before surgery	11.9 ± 4.11
Vertical AC distance (mm), day 2 after surgery	0.7
Vertical AC distance (mm) last follow-up	3.5 ± 4.1
Displacement measured on axial x-ray	
Acromio-clavciular displacement before surgery (mm), injured side	13.2 ± 10.2
Acromio-clavicular displacement (mm), last follow-up	0.91 ± 6.5
P-value between injured side at follow- up and unaffected side	p < 0.001

A significant difference was found in the CCD between pre-operative and post-operative radiographs on day two after surgery (19.1 mm± 4.18 vs. 9.0 mm± 3.84, p<0.001). At time of follow-up, the CCD had significantly increased to 12.2 mm± 3.12 (p<0.001), compared to the post-operative value (9.0 mm) and to the CCD of the contralateral uninjured shoulder (9.7 mm ± 1.84, p<0.001). For detailed analysis the results were subdivided into three groups with respect to the CCD difference to the contralateral side: <5 mm, 5-10 mm and >10 mm. There were 28 patients (85%) with a distance <5 mm, 4 patients with a distance of 5-10 mm (12%), and 1 patient with a CC distance of more than 10 mm. There was no statistical correlation between the clinical outcome (measured by the Constant score) and the radiological outcome (CC- difference to the unaffected side) with a Spearman- correlation coefficient of 0.07, p=0.83.

For the vertical AC distance, a mean of 11.9 mm± 4.11 was found for pre-operative radiographs. Two days after surgery this distance was 0.7 mm± 3.9 (p<0.001) and increased up to 3.5 mm ± 4.1 (p= 0.006) at follow-up. We found a slight over-reduction in twelve patients (12/33, 36%) at day two, but only in one patient at time of follow up.

The axial x-ray was used for evaluation of the acromio- clavicular alter displacement. When measuring the distance between the anterior edge of the clavicle and the acromion on the axial the mean distance was 13.2 mm± 10.2 pre-operatively and 0.91 ± 6.5 at follow up (p< 0.001). In 30 patients (92%) the axial view showed the anterior border of the clavicle within 1 cm ventral or dorsal of the anterior rim of the acromion.

In 10 patients (30%) we found signs of calcifications of the coraco-clavicular ligaments, but all calcifications could be graded as minor according to Dimakopoulos [18]. In 6 patients (18%) radiological signs of osteoarthritis of the AC joint occured at time of follow-up, but only one patients showed clinical signs with soreness, tenderness to palpation and positive cross body test.

There was no significant correlation between the CC calcification and the functional outcome measured by the Constant score (p=0.39), nor between osteoarthritis of the AC joint and the Constant score (p=0.38).

The average surgery time was 79 minutes (range 55–111 min). Regarding complications, an early failure of the cerclage reconstruction with complete re-dislocation was found in three patients (8%). In two cases revision surgery was performed, which revealed a knot breakage of the PDS sutures. At final follow up both patients showed excellent functional results (agCM score 95%, respectively 107%).

The third patient presenting with a CC distance of >10 mm at time of follow-up refused revision surgery due to subjective satisfaction (agConstant score of 102%).

Wound infections or nerval irritations did not occur in any of the cases.

## Discussion

The purpose of our study was to analyze the clinical and radiological results after an anatomic CC and AC cerclage stabilization for acute AC joint dislocations and we found the presented open technique for AC joint reconstruction to provide good and reliable clinical and radiological results (see Figure 2) with low complication rates.

Today, more than 80 open and arthroscopic surgical procedures have been described for the treatment of complete AC joint separations. The main goal of surgical treatment is to reduce the dislocation and create an environment for proper soft tissue healing and subsequently persistent AC joint stability. Still, reviewing the current literature, it remains uncertain, which technique provides best restoration of the AC joint anatomy and whether postoperative radiological alterations of the AC joint anatomy influence the functional outcome. One might speculate that the additional use of allografts or autografts as biological constructs provide a better long-term durability, but studies with long term follow-up are still missing. In addition, we treated only acute AC joint dislocations in our study group.

Especially techniques using a rigid fixation like the Bosworth screw [13] have failed to achieve optimal results, as the clavicle and the AC joint are highly flexible. According to Lim [27] the clavicle rises in full overhead elevation up to 35 degrees and rotates along its long axis by 45 degrees.

So the cerclage technique as a semi- rigid fixation provides a good operative treatment option for acute acromioclavicular joint dislocations and has been shown to lead to favorable clinical results [2,25,28]. Many studies used non-resorbable sutures, which are generally stiffer than resorbable sutures like PDS® (Ethicon, Norderstedt/ Germany) and might therefore lead to stress fractures of the coracoid process as described in literature [29]. Anyway, we did not find any fractures or erosions of the coracoid process in the evaluated patients. However, being aware of the material properties of PDS® sutures [21-23], an anatomic or at the utmost vertical over-correction of 3 mm at time of surgery resulted in an almost anatomic reduction at time of follow-up. 28 patients of the radiologically examined 30 patients (93%) had a CC difference of up to 5 mm compared to the contralateral side. Nevertheless, we had 5 patients with insufficient reduction and two of them had to undergo early revision surgery due to knot breakage. The remaining secondary failures can by explained by the elongation of the resorbable sutures.

However, biomechanical cadaveric studies showed that the cerclage techniques lead to an anterior displacement of the clavicle [30,31], being one of the major disadvantages of this technique. So we used an anatomic AC joint reconstruction with two CC cerclages and one AC cerclage to allow for clavicle rotation, but limit the anterior displacement. We could show that by the use of the additional AC cerclage the horizontal displacement could be reduced and that in 30 of 33 patients the anterior rim of the clavicle was within 10 mm anterior or posterior of the acromion. These results correlate well with a cadaver study by Beitzel et al. [32], who found in the native shoulder specimen a mean anterior translation of the clavicle of 7.92 mm± 1.69 mm and a posterior translation of 7.84 mm± 2.09 mm when applying a load of 70 N.

Radiologically, the rate of AC osteoarthritis or degenerative alterations was 19%, and the rate of calcifications of the CC ligaments was 30%. The lower radiological alterations seen in the present study compared to the study of Greiner et al. [2] (rate of calcifications: 68%, radiologic signs of osteoarthritis: 74%) might be due to the shorter follow-up in the present study (32 months vs. 70 months). However, Dimakopoulos et al. [18] reported no signs of osteoarthritis in their cohort after double-cerclage reconstructions of the CC-ligaments at a similar follow-up of 33.2 months. Therefore, the decisive factors for development of these changes need to be defined.

Ladermann et al. [3] used a double cerclage technique with non-absorbable sutures equal to the present technique and found that patients with a CC distance <5 mm showed significantly better results in the Constant Murley score and the DASH score in comparison to patients with a subluxated AC joint (P < .005). This could not be supported by our study, however the number of patients having CCD > 5 mm (n=2) was too small to ensure statistical power.

Although a postoperative increase of the CC distance was noticed in 73% of the patients, the overall clinical results were good to excellent. This finding supports former studies showing that a residual AC dislocation does not imperatively lead to complaints or loss of strength [2,9,33], so that the radiological results appear to have minor correlation to clinical outcome.

Operative treatment of acromioclavicular joint dislocations with the described cerclage technique is a safe AC reconstruction technique: The AC and CC ligaments are reconstructed anatomically, the technique is easy to handle, provides good to excellent clinical results as shown by the clinical scores and the use of absorbable material makes a hardware removal unnecessary.

Furthermore, the AC discus can be examined or removed if necessary and the reconstruction of the delto-trapezoideal faszia can be performed, particularly in type V injuries.

Nevertheless, in the past few years all-arthroscopic techniques have emerged and several studies have shown good to excellent clinical and radiological results [4,34], though the important deltoideo-trapezoidal fascia is not addressed by these techniques [10].

The major advantage of these arthroscopic procedures is that concomitant shoulder injuries can be detected and addressed at the same time if necessary. In a study by Tischer et al. [35] 18% of patients with AC joint dislocation grade Rockwood III-V had intra-articular pathologies such as SLAP lesions or injuries of the rotator cuff. However, complications caused by an overly marginal placement of the tunnels in the coracoid process leading to a dislocation of the tight rope**®** (Arthrex, Naples/USA) or a fracture of the coracoid process [29] have been described, so that this technique is reserved to experienced shoulder arthroscopists.

The present study has several limitations, including a relatively small number of patients in each Rockwood type subgroup with a subsequent decrease in power as well as its retrospective and non-randomized design. A long-time follow-up would be needed to finally determine the incidence of posttraumatic alterations of the AC joint.

Nevertheless, the study has the strength of assessing seven outcome measurements including Constant score, DASH score, ASES score, VAS, Simple Shoulder Test, physical examination findings and radiographic evaluation in two planes in a suitable number of treated patients.

## Conclusion

The described technique turned out to be an interesting alternative to conventional AC reconstruction techniques given the improved anatomic reconstruction as well as its simplicity and reproducibility. The clinical results were good to excellent and radiological results showed an almost anatomic reconstruction. Radiological postoperative changes like the increase of CCD over time, occurrence of AC osteoarthritis or CC calcifications did not have significant influence on the functional outcome at final follow up.

Future research needs to evaluate the effects of the additional cerclage in the long term. Due to the tendency of PDS® to stretch after surgery, we recommend a slight over-correction of 3 mm in the horizontal plane and a restrictive rehabilitation scheme with limited range of motion to 90° in abduction and flexion for six weeks.

## Competing interests

The authors declare that they have no competing interests.

## Authors' contributions

All authors contributed in a significant way in the steps of writing and editing the manuscript. GHS and FM conceived of the idea for the study/publication and engaged in writing the first draft. LM carried out the outcome evaluation. TMK was involved in the statistical analysis and provided research support. AB provided expertise in artwork. TF and PA provided additional research support and were involved in the manuscript writing. US and SS gave advice throughout the project and reviewed the manuscript. All authors read and approved the final manuscript.
